# The Impacts of Hinged and Solid Ankle-Foot Orthoses on Standing and Walking in Children with Spastic Diplegia

**Published:** 2013

**Authors:** Hamid DALVAND, Leila DEHGHAN, Awat FEIZI, Seyed Ali HOSSEINI, Susan AMIRSALARI

**Affiliations:** 1 Department of Occupational Therapy, University of Social Welfare & Rehabilitation Sciences, Tehran, Iran; 2 Department of Biostatistics and Epidemiology, School of Health, Isfahan University of Medical Sciences, Isfahan, Iran; 3 New Hearing Technologies Research Center, Department of Pediatric Neurology, Faculty of Medicine, Baqiyatallah University of Medical Sciences, Tehran, Iran

**Keywords:** Child, Cerebral palsy, Spastic diplegia, Equinus deformity, Orthosis, Rehabilitation

## Abstract

**Objective:**

The purpose of this study was to examine the impacts of hinged and solid anklefoot orthoses (AFOs) on standing and walking abilities in children with spastic diplegia.

**Materials & Methods:**

In a quasi-experimental design, 30 children with spastic diplegia, aged 4-6 years were recruited. They were matched in terms of age, IQ, and level of GMFCS E&R. Children were randomly assigned into 3 groups: a hinged AFO group (n=10) plus occupational therapy (OT), a solid AFO group (n=10) plus OT, a control group who used only OT for three months. Gross motor abilities were measured using Gross Motor Measure Function (GMFM).

**Results:**

We obtained statistically significant differences in the values between baseline and after treatment in all groups. The groups were also significantly different in total GMFM after intervention. Furthermore, there were differences between hinged AFOs and solid AFOs groups, and between hinged AFOs and control groups.

**Conclusion:**

We concluded that gross motor function was improved in all groups; however, hinged AFOs group appears to improve the gross motor function better than solid AFOs and control groups.

## Introduction

Standing and walking serve an individual’s basic need to move from place to place and both are the most common activities that people do on a daily basis. In children with cerebral palsy (CP), lesion in central nervous system (CNS) could cause motor-sensory impairments that progressively deteriorate over time (1). Loss of ability to move and manage the environment can result in loss of self-esteem and deep sense of dependence. 

Equinus deformity is the most commonly recognized ankle joint malposition in children with CP (2). Wren et al. (2005) reported that equinus deformity was the most prevalent deformity among the 14 specific gait abnormalities, and seen in more than 50 % of children with spastic diplegia (2).

Ankle-foot orthoses (AFOs) have been administered for foot deformities in children with CP (3). A systematic review suggested the use of AFOs for improvement of foot and ankle deformities, and as a result enhanced normal gait pattern (i.e., from toe walking or flat foot strike to heel-strike) (4).

Brehm et al. (2008) investigated the effects of AFOs on walking efficiency and gait in a heterogeneous group of children with CP. The use of AFOs resulted in significant decrease of energy consumption during walking in children with quadriplegic CP, compared to barefoot walking, whereas it remained unchanged in hemiplegic and diplegic children. The improvements of efficiency were reflected in changes of stance and swing phases of knee motion, (i.e., children whose knee flexion angle improved toward the typical normal range showed a decline in energy consumption) (5). 

The AFOs include many different types (e.g., solid, hinged), and almost all published studies have confirmed the mechanical effects of these orthoses (6-12).

Although several studies reported positive effects of hinged AFO (HAFO) in kinematic and kinetic analysis of gait of children with CP (7-9), some studies found no difference between HAFO and SAFO. Both kinds of orthoses increased the stride length, reduced cadence, and decreased excessive ankle plantar flexion compared with no orthosis (9-12), and as a result, had positive effects on stance balance (13). Over the past two decades, interest has been emerged in using functional assessments in neurodevelopmental disability at clinical setting. These assessments are reliable, valid, available, and easy to use. Among these assessments, the Gross Motor Function Measure (GMFM) has been used in several studies (14-17) with controversial results. In two studies, GMFM was not sensitive enough to assess the effects of HAFO and dynamic AFO treatments, although, both orthoses were equally effective in improving the ankle kinematics and kinetics in children with diplegic CP (15,16). However, another study showed significant improvements in range of motion, Berg Balance Scale, and GMFM scores in the standing and walking abilities in two groups (dynamic DAFO and solid SAFO) of children with spastic diplegia (14,17). The importance of the choice of appropriate AFOs (i.e., hinged and/or solid) in improvements of standing and walking in these children are quite critical. When an orthotic is given correctly, the participant will perform ADLs better and more independently. Walking and standing functions have significant impact on activities of daily living of children with CP. Furthermore, there is an inconsistency in the results of studies about the effects of HAFO and SAFO on gross motor function (standing and walking) of children with spastic diplegia. Hence, this study was conducted to evaluate and compare the effects of hinged and solid AFOs on standing and walking abilities in children with spastic diplegia.

## Materials & Methods

The present research was a quasi-experimental study with pre-/post- design. Thirty children with spastic diplegia, aged 4 to 8 years, randomly allocated into 3 equal groups. The children were referred by a pediatric neurologist to the rehabilitation clinic affiliated to the University of Social Welfare and Rehabilitation Sciences (USWR). All procedures were approved by the Medical Ethics Committee (MEC number 801/4/88/1233) at USWR in Tehran in the spring of 2011 and the parents of patients were informed about the design of the study and wrote informed consents were also obtained.

**Instrumentation and protocol**

The participants were selected through convenience sampling strategy. Children were matched in terms of age, intelligence quotient (IQ) and level of Gross Motor Function Classification System Expanded & Revised (GMFCS E&R), and then randomly allocated into 3 equal groups. Each group of patients was randomly assigned into one of the therapy methods, in which these groups consisted of hinged AFOs, solid AFOs and barefoot (control group). All three groups received neurodevelopmental treatment (NDT) for 3 months (3 sessions per week, 1 hour daily) by an occupational therapist that was not in the research team (i.e., had no idea about the grouping of children) (Flow chart 1). 

NDT is not purely a treatment technique but it is a way of observing, analyzing a child’s performance, and finding his/her potential abilities. The main purpose of this approach is to correct the abnormal postural tone and to facilitate the normal movement patterns for the performance skills (18).

**Flow chart1:**
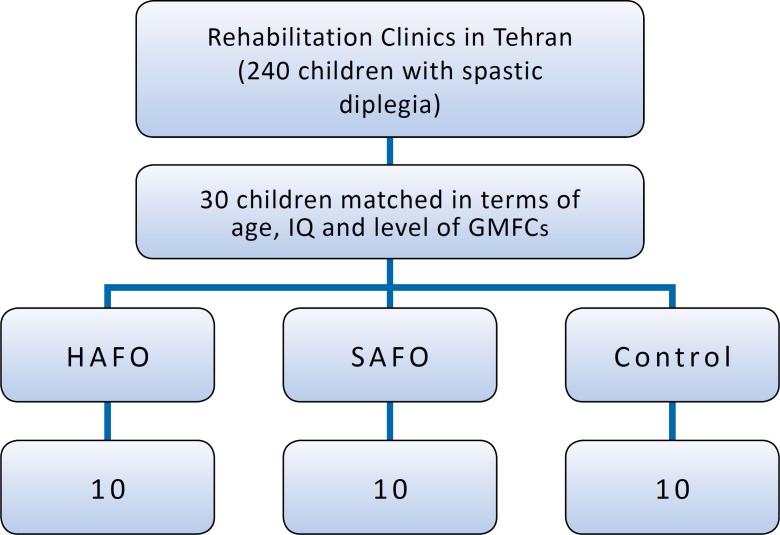
Study flow chart. A total of 240 children with Cp were enrolled; 30 children matched in term of age,IQ and level of GMFCS, Ten children included in each group and each group of patients was randomly assigned into one of the therapy methods in which these groups consisted of HAFOs, SAFOs and barefoot (control group).

Children were examined by pediatric neurologist and cases with normal IQ; ability to stand and walk with an assistive device or independently; no history of wearing any hinged and or solid AFOs; no seizure and or epilepsy; no congenital dislocation of hip (CDH); no history of surgical procedures; no Botulinum injections within the last 9 months on the lower extremity; level I to III of GMFCS E&R; no pathologic crouch gait; and/ or apparent equinus were included in the study.

The following assessment protocol was carried out in all subjects by professionals who were blind to children’s allocation. 

Severity of CP was solely based on gross motor function as judged by therapists using the GMFCS E&R, which is a reliable and a valid five-level pattern recognition classification system that differentiates the children with CP, according to their age specific gross motor activity (19,20). The GMFCS E&R describes the major functional features of children with CP in each level within several age ‘windows’: before their second birthday, between age 2 years and the fourth translated into Persian by Dehghan et al. in 2011, and it indicated good reliability (21).

Abilities of gross motor function were assessed using GMFM-88 by an occupational therapist. GMFM-88 is a clinical tool designed to evaluate changes in gross motor function in children with CP. GMFM-88 consists of a spectrum of activities, ranging from lying and rolling up to walking, running and jumping skills. GMFM-88 is appropriate for children whose motor skills are at or below those of a 5-year-old child without any motor disability (22). The results of a study in 2005 suggested that the GMFM-88 might provide a clinically useful tool to help in understanding the impact of orthoses on gross motor skills of children with CP (23). In present study, according to the GMFM-88 manual, only part “D” and “E” that respectively assess standing and walking, were used.

**Fig 1:**
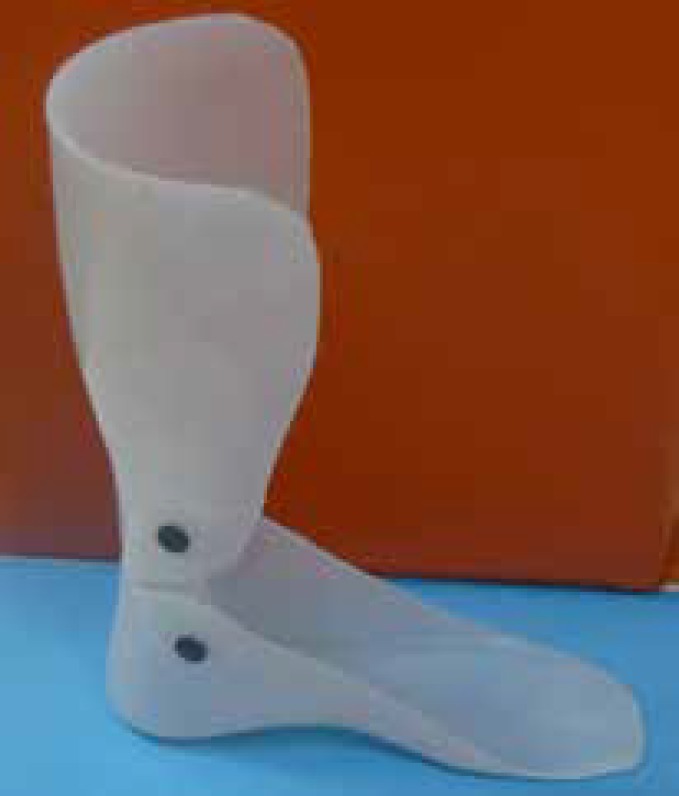
Hinged AFO

**Fig 2:**
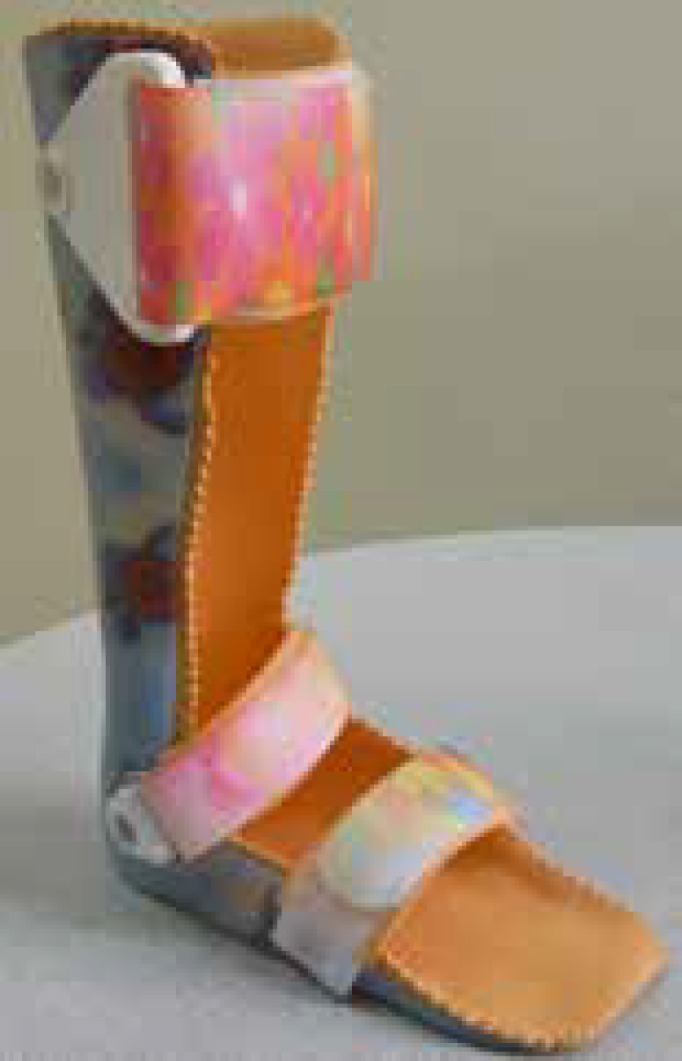
Solid AFO

The HAFO and SAFO were custom-made for each subject from the same positive mold after casting the lower extremity by an orthotist at the clinic (Figure 1 and 2). Both orthoses were fabricated from 3.0 mm thick propylene, extending distally under the toes and proximally on the posterior surface of the leg to about 2.5-5 cm below the knee. The HAFO articulated with 90 °plantar flexion stop and free dorsiflexion. The SAFO fixed the ankle in a neutral position without any movement. Subjects were instructed to wear AFOs regularly for 3 months, 6 hours daily (42 hours per birthday, between age 4 years and the sixth birthday, and between age 6 and 12 years. Table 1 outlines the main abilities of children aged 4 to 6 and 6 to 12 years in each GMFCS E&R level. Use of the GMFCS E&R requires familiarity with the child, but requires no formal training. This classification system was already week). All children in hinged and solid AFOs groups attended all 36 training sessions and there were not problems fitting and using braces. 

**Data analysis**

All data were collected before and after intervention.

The results (i.e., within groups comparisons) were analyzed using Wilcoxon signed rank statistical test. 

Between groups comparisons (i.e., after intervention results, the differences in improvement of gross motor function scores) were analyzed using Kruskal-Wallis and Mann-Whitney U tests.

## Results

Descriptive characteristics of the participants were summarized in Table 1. There was no significant difference in the means of age (p=0.2) and IQ (p=0. 9) across the groups.

It can be seen from Table 2 that there was a significant difference between total gross motor function scores before and after intervention for HAFOs group, for SAFOs, and for the control group (p<0.01). A significant difference was found among the three groups in gross motor function using the Kruskal-Wallis test (p<0.01; Table 3).

Mann-Whitney U test, adjusted for type I error, and it was used as a post hoc procedure after Kruskal-Wallis.

Results of Mann-Whitney U test showed a significant difference when comparing hinged AFOs group with solid AFOs (p<0.05) and the control groups (p<0.01) in improvement of gross motor function, but there was no significant difference between solid AFOs group and the control group (p=0.631; Table 4).

## Discussion

The results of the present study showed significant improvement in standing and walking functions in children with spastic diplegia after application of HAFO using GMFM. The GMFM reflects the motor developmental sequence from birth to 5 years, it necessarily includes activities that precede or that are prerequisites for the achievement of gait (24). A greater range of functional gait-related activities can be assessed using the GMFM. Findings with regard to the HAFO group in this study were consistent with several other studies (15,16,25). HAFO showed the greatest improvements in gross motor function (i.e., walking/ running/jumping dimensions of GMFM) (15,25).

This might be due to that provided by HAFO free dorsiflexion in the stance phase and limited plantar flexion (usually 90°) that normalizes ankle motion during the stance phase of gait and facilities the performance of developing motor skills (8,26,27). Accordingly, the use of HAFO, specifically a configuration that enabled ankle dorsiflexion, enhanced coordination, facilitated a more normal weight shift in all planes, improved symmetry, and therefore, smoothed the gait pattern (25).

Furthermore, this suggestion has been shown by electromyographic and kinematic data, in which HAFO has more advantages for children with spastic CP (9). 

The peak activity of the tibialis anterior muscle was reduced at initial contact and loading response phase and just after toe-off, when using a HAFO. The reduction in activity was assumed to cause by the change in gait pattern from a toe-gait to a heel-toe gait as well as by use of a HAFO (9).

Normal ambulation requires approximately 10 degrees of dorsiflexion and 20 degrees of plantar flexion. Any limitation of motion at the ankle leads to an abnormal gait pattern (28). Dorsiflexion in swing phase has two roles: first, in early swing phase, helps to shorten the limb and allows swing through; and second, in terminal swing phase, dorsiflexion is part of prepositioning the limb for initial contact. On the other hand, most children with CP in level between I to III of GMFCS E&R, have a fairly good motor control at the hip and knee joint and in fact they need some help for their distal motor control at the ankle joint.

However, a study on diplegia (ranging 4 years and 4 months to 11 years and 6 months) showed a detrimental effect on function with HAFO use (29). This result might be due to the mean age of the study sample, as Rodda et al. (2004) suggested that apparent equinus and crouch gait were seen in children who were 2.9 years older than those with true equinus and jump gait (30). It means that with the increase of age, the pattern of gait shifted from true equinus and jump gait to apparent equines and crouch gait. Therefore, because of the ankle normal position in apparent equinus and ankle dorsiflexion in crouch gait, use of HAFO cannot improve the standing and walking functions.

It seems that the application of HAFO could help these patients during the terminal stance via increased ankle dorsiflexion and also through increased ankle plantar flexion and power generation during the pre-swing phase. Accordingly, HAFO might be more appropriate to prescribe for children with spastic diplegia, (aged 4-6 years old); however, further individual factors (e.g., type of CP and level of GMFCS E&R) should be considered when selecting orthosis.

Our study had a few limitations: first, we had a few samples in each group due to the convenience sampling method; second, in this study, we used low-tech clinical tools (GMFM-88), because it was not possible to access high-tech tools. 

Based on the results of this study, we concluded that HAFO can significantly improve standing and walking functions in children with spastic diplegia. 

This in respect might be useful to prescribe HAFO for rehabilitation program of children with spastic diplegia.

**Table 1:** Descriptive Characteristics of The Study Groups

		**HAFOs**	**SAFOs**	**Control**
**Age (months; mean±SE)**		78±5.307	71.20±4.017	67.83±4.764
**IQ (mean±SE)**		94.25±1.377	98.80±1.281	96.33±3.20
**Gender**	Male	3	5	5
	Female	7	5	5
**GMFCS Levels**	I	3	4	5
	II	5	4	4
	III	2	2	1

**Table 2:** Wilcoxon Signed Rank Test Results For Comparing Total GMFM Scores Before and After Intervention in Each Group

Group	Average GMFM score before	Average GMFM score after	Z	p-value
HAFOs	26.12	33.97	-2.803	0.005*
SAFOs	29.88	35.43	-2.803	0.005*
Control group	30.68	35.69	-2.805	0.005*

* p<0.01 , Z: Normal Distribution

**Table 3:** Kruskal-Wallis test’s result for comparing of mean differences of total GMFM scores before and after intervention among three groups

Group	HAFOs	SAFOs	Control	χ^2^	p-value
statistic					
Mean rank difference of GMFM score before-after intervention	21.70	13.45	11.35	8.143	0.017

**Table 4:** Mann-Whitney U test’s results for comparing of mean differences of total GMFM scores before and after intervention between two considered groups

Group	HAFOs with SAFOs	HAFOs with Control	SAFOs with Control
p-value	0.034*	0.007**	0.631

** p<0.01,

* p<0.05
